# Maintaining quality newborn care in Ghana amid the COVID-19 pandemic

**DOI:** 10.11604/pamj.2020.35.4.22797

**Published:** 2020-04-16

**Authors:** Alhassan Abdul-Mumin, Faith Agbozo, Abdulai Abubakari, Albrecht Jahn

**Affiliations:** 1 Department of Pediatrics and Child Health, School of Medicine and Health Sciences, University for Development Studies, Tamale, Ghana; 2 Department of Pediatrics and Child Health, Tamale Teaching Hospital, Tamale, Ghana; 3 Department of Family and Community Health, School of Public Health, University of Health and Allied Sciences, Ho, Ghana; 4 Department of Public Health, School of Allied Health Sciences, University for Development Studies, Tamale, Ghana; 5 Heidelberg Institute of Global Health, Heidelberg University Medical Faculty, Germany

**Keywords:** COVID-19 pandemic, newborn care, Tamale, Ghana

## To the editors of Pan African Medical Journal

The novel Coronavirus (2019-nCoV) outbreak and its associated illness (COVID-19), characterized by life-threatening respiratory distress, was first reported in Wuhan China in December 2019 [1]. The World Health Organization (WHO) declared it a Public Health Emergency of International Concern in January 2020 and subsequently, a pandemic in March 2020 [2]. The virus has affected almost all countries and territories causing 1,653,204 infections globally and 12,952 infections in Africa as of 11th April 2020 [3], thus becoming the most important public health problem since the beginning of the 21st century. Ghana confirmed its first two cases on 12th March 2020. Although 105 cases were intenational travelers who underwent mandatory quarantine, of the 408 cases confirmed as of 11th April 2020 [4], 57.1% are likely due to community transmission detected through enhanced contact tracing and routine surveillance [4]. Most recently, cases have been reported in persons below 15 years, but the exact age distribution among this group is not publicly known. So far, cases have been reported in eight of the 16 administrative regions of Ghana. In the Northern region, ten cases were confirmed among West African nationals who travelled into the country in March.

Globally, among the pediatric population, reports have shown that prevalence is lower, the median age of infected children is about seven years, but infants are more vulnerable, the clinical course is milder, and their case fatalities are fewer even in the presence of underlying morbidities [5,6]. A few cases have been reported in the newborn period all of which are associated with maternal history. Till now, there is no evidence of intrauterine or transplacental transmission from infected pregnant women to their newborns [7]. Howeer, a recent report in the United States of America linked the death of a six-week old infant to complications of COVID-19 [8]. In line with recommendations from the WHO, measures have been instituted by the Ministry of Health and Ghana Health Service to curtail the spread of the virus. The Tamale Teaching Hospital (TTH) is for now, the only designated treatment center serving the Northern region of Ghana. Regarding neonatal care in TTH, there is a 50-bed capacity Neonatal Intensive Care Unit (NICU) and an additional five-bed Kangaroo Mother Care unit. The unit is the only referral facility providing specialized care for sick neonates in the catchment population of more than four million in northern Ghana. The policy not to turn away any patient has led to congestion in the unit with the daily ward state generally between 60-70 neonates.

We briefly describe the institutional and departmental precautions taken in the wake of the COVID-19 pandemic to ensure ´physical distancing´ and how it is already impacting on newborn care services. As a treatment center, the facility is undergoing decongestion and clients are advised not to visit the hospital unless it is really necessary. Our weekly specialist outpatient clinic where an average of 40 high risk neonates including babies born preterm, hypoxic ischemic encephalopathy, severe jaundice and congenital anomalies are reviewed has been suspended. All teaching and internship programs by medical and nursing students have also been suspended. Also, we have relocated our daily NICU clinic where we see referred sick neonates to the hospital´s general outpatient´s department, which is a substantial distance away from the NICU. In addition, we have banned all forms of visits by patients´ relatives and restricted entry to only a limited number of mothers at a time to breastfeed or send expressed breastmilk. We have also offered refresher training on Infection Prevention and Control (IPC) measures to staff and have provided additional handwashing and sanitization opportunities in and around the unit for both staff and parents. Restriction of entry for parents defeats the drive to promote family-centered care in the NICU and will impede bonding between the mother and baby. As the Nation and institutions divert resources towards COVID-19 response, we envisage shortage of supplies for essential newborn care. In our society where healthcare seeking behaviors are sub-optimal, and neonatal mortality constitutes about 50% of under five deaths [9], we fear that gains made in our newborn care program might be derailed as result of the pandemic.

The provision of more handwashing and sanitization opportunities at the NICU and refresher training of staff on IPC practices is one positive impact likely to emanate from this pandemic. This will be key in our efforts to reduce health care associated infections even beyond the pandemic. The COVID-19 pandemic although unprecedented, has not only rekindled adherence to IPC protocols in newborn care but also provided insights on actions to decongest our overcrowded unit. In order to mitigate the negative effect of our inability to continue with routine review clinics during this difficult period, we shall explore the feasibility of home-based mobile-health and telemedicine technologies in addition to improved client education to ensure continuity of care for our vulnerable newborns. In doing this, we shall draw on the Ghana Health Service guideline on home and community-based treatment of neonatal infections [9]. While we generate the evidence, we can only hope that impediments with mobile networks particularly in rural areas, unavailability of smartphones, poverty, illiteracy and lack of funding for research are surmounted.

## Conclusion

Although Ghana has recorded few cases in children <15 years since the first COVID-19 cases were confirmed in March, restrictions imposed to contain the spread is already impacting on the newborn care service delivery at the TTH. Neonatal mortality indirectly related to COVID-19 might increase if the pandemic lingers longer. Instead of waiting to assess the scale of impact of the pandemic, damage control measures like improved home-based continuum of care, equipping families to contribute to the newborn care process complemented with m-health approaches are necessary now.

## Competing interests

The authors declare no competing interests.
